# Wharton’s Jelly Tissue Allografts for Tearing in the Plantar Fascia: A Case Series

**DOI:** 10.3390/biomedicines13102328

**Published:** 2025-09-24

**Authors:** Babak Baravarian, Gi Kwon, Conrad Tamea, John Shou, Naomi Lambert, Alexis Lee, Eva Castle, Tyler Barrett

**Affiliations:** 1 University Foot & Ankle Institute, Santa Monica, CA 90403, USA; bbaravarian@mednet.ucla.edu (B.B.); gi.kwon@footankleinstitute.com (G.K.); 2 Orthopedic Associates of Tampa Bay, Tampa, FL 33603, USA; conradtameamd@gmail.com; 3 Regenative Labs, Pensacola, FL 32501, USA; drshou@regenativelabs.com (J.S.); alexis@regenativelabs.com (A.L.); eva@regenativelabs.com (E.C.); tyler@regenativelabs.com (T.B.)

**Keywords:** plantar fasciopathy, plantar fascia, plantar fasciitis, foot neuropathy, wharton’s jelly, regenerative medicine

## Abstract

**Introduction**: Plantar fasciitis (PF), or more recently plantar fasciopathy due to its degenerative nature, is the most common cause of heel pain in adults and is often refractory to conservative care. One alternative conservative intervention involves replacing damaged fascia with homologous tissue, such as Wharton’s jelly (WJ) connective tissue allografts. The purpose of this observational study is to evaluate the efficacy and safety of collagen-rich Wharton’s jelly (WJ) when applied to defects in the plantar fascia. **Materials and Methods**: From the observational repository, nine patients who had plantar fasciopathy and received a single application of WJ were observed over 90 days. Outcomes were tracked using the Numeric Pain Rating Scale (NPRS), the Western Ontario and McMaster University Arthritis Index (WOMAC), and the Quality-of-Life Scale (QOLS) with no adverse reactions reported. **Results**: The cohort was 56% male (*n* = 5) and 44% female (*n* = 4), with a mean age of 73. From the initial to final visit, patients reported an overall trend of improvement in all scales. Statistically significant Bonferroni-adjusted differences were observed in the WOMAC scale. Age was a significant predictor of the total WOMAC score change from the initial to the final visit. **Discussion**: Although only a small cohort was observed, the preliminary evidence suggests the safety and efficacy of WJ allografts for plantar fascia degeneration. Key limitations of this study included a small cohort size and a lack of a comparison group with other alternative methods. **Conclusions**: The favorable results in this study could encourage future research to validate the clinical potential, safety, and dosing protocols of Wharton’s jelly as a primary conservative care method for patients suffering from plantar fasciopathy.

## 1. Introduction

Plantar fasciitis is the most common cause of heel pain in adults, accounting for approximately 1 million annual clinical visits in the United States [1,2,3]. While commonly known as plantar fasciitis, a shift away from the nomenclature has been made over the past 20 years, as research indicates it is not an inflammatory condition. Plantar fasciosis (PF), or plantar fasciopathy, more accurately describes what imaging studies have demonstrated to be degeneration of the plantar fascia in the form of micro-tearing in the collagen structures at the insertion site and along the fascia [4,5]. PF affects approximately 10% of the general population, irrespective of age or physical activity levels [1,6]. Several medical issues, encompassing neurological, arthritic, traumatic, neoplastic, infectious, or vascular factors, may result in the occurrence of plantar enthesopathy [6]. Risk factors, patient history, and physical examination are used to guide the diagnosis of PF. Common risk factors include elderly age, high BMI, and female gender [7]. A study by Liu et al. (2024) showed that a decline in calcaneal elasticity present under mechanical stress leads to microfractures and inflammation in the fascia and surrounding muscle groups, contributing to heel pain [8]. Continuous strain or repetitive impacts can cause both micro and larger tears in the plantar fascia [9]. These tears are the primary cause of patient pain. If the strain on the fascia persists, it can prevent the body from facilitating effective repair.

Because the exact cause of plantar fasciopathy is not well established, several conservative and invasive interventions are available. Conservative options include lifestyle modifications, plantar fascia-specific stretching, orthotics, NSAIDs, and focal extracorporeal shockwave therapy [10]. Orthotics and lifestyle changes in footwear have been a common management technique for PF patients; however, while these strategies might improve pain, evidence indicates that their long-term effectiveness is limited, with studies demonstrating only short-term improvement, indicating that they primarily serve to delay, rather than prevent, symptom recurrence [11,12]. In addition to footwear changes, NSAIDs are another common approach to PF. According to a study by Nahin (2019), around 50% of individuals with PF in 2013 used over-the-counter NSAIDs [13]. Between 2010 and 2018, the number of plantar fasciopathy (PF) cases more than doubled, and this trend is expected to persist, thereby escalating the annual economic burden. Each year, nearly $600 is spent on NSAIDs per individual. When factoring in the costs of standard treatments, the total annual expenditure for PF amounts to $284 million [14]. Conservative treatment options may provide temporary symptomatic relief, but the underlying tissue damage remains unaddressed. While invasive options are available, surgery is often a last resort in plantar fasciopathy, typically for patients who do not respond to conservative interventions for at least 6 to 12 months [15]. Multiple complications may arise following surgical interventions, with pain recurrence being the most prevalent [16]. Other complications include nerve injury, plantar fascia rupture, and flattening of the longitudinal arch [15]. With the risk of unfavorable outcomes post-surgery and a potential rise in pain levels, it is evident that there is an urgent need for alternative care. The rise in autologous platelet-rich plasma therapy as a regenerative intervention has been considered a popular replacement for current standard-of-care methods. Caution is necessary when interpreting the results of studies on PRP injections, as numerous investigations have shown no notable differences between the group receiving PRP and the placebo. A study by Johnson-Lynn et al. (2018) observed no significant benefit for the use of PRP over normal saline in the management of PF [17]. As reported by Valotto-Junior et al. (2023), there were no meaningful differences in the quality of life, and the presence of platelets did not influence pain alleviation [18]. Establishing reliable and beneficial methods for the management of PF that target the structural degradation of the fascia is essential for the continued development of conservative care options in patients suffering from treatment-resistant PF.

Current investigation into alternative approaches has been encouraged due to the growing concern for better management of PF. One emerging conservative care method is Wharton’s jelly (WJ) tissue allografts: a human umbilical cord-derived connective tissue that consists of a collagenous-rich matrix with collagen types I, II, III, IV, V, VI, XII, and XIV as well as other extracellular matrix (ECM) components, including proteoglycans, glycosaminoglycans, fibronectin, fibrillin, and hyaluronic acid [19,20,21]. The structural components of WJ are comparable to the ECM of human articular cartilage, tendons, enthesis, and dermal tissues [22]. WJ has been applied to over 180 homologous use sites, indicating its safety and efficacy as a homologous tissue allograft [23]. While there has been limited research published on the outcomes of WJ applications in PF patients, WJ tissue allografts can be transplanted to supplement the damaged fascia and minimize the adverse effects of plantar fasciopathy. Considering the limited conservative options for the management of plantar fasciopathy, this observational research study aims to evaluate the safety and clinical potential of Wharton’s jelly supplementation for defects of the plantar fascia.

## 2. Materials and Methods

### 2.1. Study Design

**Figure 1:**
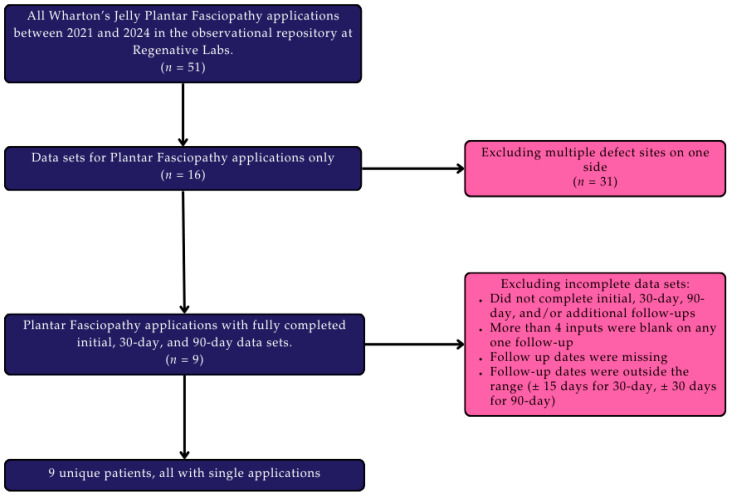
Study Design Flow Chart.

Data were obtained from the observational repository at Regenative Labs following the guidelines of the Declaration of Helsinki with approval from the Institutional Review Board of the Institute of Regenerative and Cellular Medicine (IRCM-2021-311). Informed consent was collected from all patients in this study. The repository design and database have been described in detail in previous publications [23]. Participants identified from the observational repository included patients with complete initial, 30-day, and 90-day data sets with plantar fascia-specific defects. Excluded patients included those who were lost to follow-up, those with missing data, and data sets that were outside of the respective time range (±15 days for 30-day follow-up, ±30 days for 90-day). Figure 1 displays a visual representation of this study’s design as well as the inclusion and exclusion criteria. Regenative Labs, Pensacola, FL, USA, adheres to the guidelines set by the FDA and American Association of Tissue Banks (AATB) in its tissue processing protocols. Additional manufacturing information is available on their website.

### 2.2. Patient Population

Patients presented with post static dyskinesia at their initial consultation. The plantar fascia was then checked with ultrasound for structural defects, including thickening hypoxic areas, osteophytes, tissue damage, and micro-tearing. This evaluation confirmed the diagnosis of plantar fasciopathy with evidence of fascia degeneration. Patients were prescribed conservative care for at least three months, including physical therapy, massage, shoe modifications, anti-inflammatories, home exercises, and orthotics. The patients in this group all failed at least three months of these conservative care options and opted for Wharton’s jelly allografts before considering more invasive interventions. All patients received only one allograft application. Patients selected for this study ranged from 50 to 89 years of age, with a mean age of 73 years. Patient Body Mass Index (BMI) ranged from healthy weight (18.5–24.9) to obese (>30.0). Three patients opted out of reporting BMI. Four females and five males participated in this study. Patient demographics are displayed in Table 1.

### 2.3. Patient Care Procedures

On the day of the procedure, tissue degeneration was identified via ultrasound, and 2 cc of 1% lidocaine, which was the physician’s choice of anesthetic, was injected into the specific points of deterioration to confirm that the site of pain was caused by the micro tears in the patient’s fascia. If the patient confirmed a decrease in pain after the lidocaine injection, the area of tearing was then filled in with 2 cc of 150 mg/mL Wharton’s jelly (WJ) allograft (ProText™, Regenative Labs, 1700 W Main St., Pensacola, FL 32502, USA) via a 25-gauge needle under ultrasound guidance. Micro-tearing and fascial degeneration most often occurred at the insertion site or slightly distal to it. A medial approach was used to place the WJ allograft into the fascia at the insertion site and distally along visible degeneration. Following the procedure, the patient was instructed to wait at the office for thirty minutes to be monitored for any signs of a reaction. No patients reported adverse reactions throughout the observational period. Patients filled out the Quality-of-Life-Scale (QOLS), Numeric Pain Rating Scale (NPRS), and Western Ontario and McMaster Universities Osteoarthritis Index (WOMAC) scales on the day of the application, and again 30 and 90 days after. Aftercare procedure instructions included massaging the area with the thumb to reduce and break up scar tissue, massaging the medial tubercle and heel area twice a day, restricting the use of anti-inflammatories and ice for six weeks, and stretching the Achilles and calf muscles twice daily.

### 2.4. Data Analysis

Descriptive statistics for the patient cohort, including mean, median, standard deviation, skewness, and kurtosis, were computed for baseline comparison. Patient-reported outcomes were measured using the NPRS, WOMAC (Pain, Stiffness, Physical Function subscales, and Total), and QOLS at initial and follow-up visits. The improvement was determined by calculating the percentage change from initial to 30-day and 90-day follow-ups. Nonparametric analysis was utilized to test for any statistical relevance in the improvement of scores over time using the Wilcoxon Signed Rank Test (WSRT) due to the small sample size and the data distribution. For each scale in the WSRT, three pairwise comparisons were tested. To control Type I error within each outcome, Bonferroni correction was applied within each family of three comparisons (adjusted α = 0.05/3 = 0.0167). Statistically significant differences were bolded in the tables below. Demographic factors such as age, BMI, and gender were tested to identify significance in overall total WOMAC score changes; however, age and gender were only included due to sample size and missing data in BMI. Potential confounders were adjusted with Bonferroni and bootstrapping. The correlation coefficient, r, was calculated only for significant values to indicate effect size. No imputation was performed. All statistical tests were two-tailed, with a significance threshold set at a 95% CI, *p* < 0.05 (except for WSRT). Although the cohort was divided by gender for comparison, no statistical analysis was performed due to insufficient sample size. Analyses were performed using SPSS Statistics (Version 30, IBM Corp, Armonk, NY, USA).

## 3. Results

**Figure 2:**
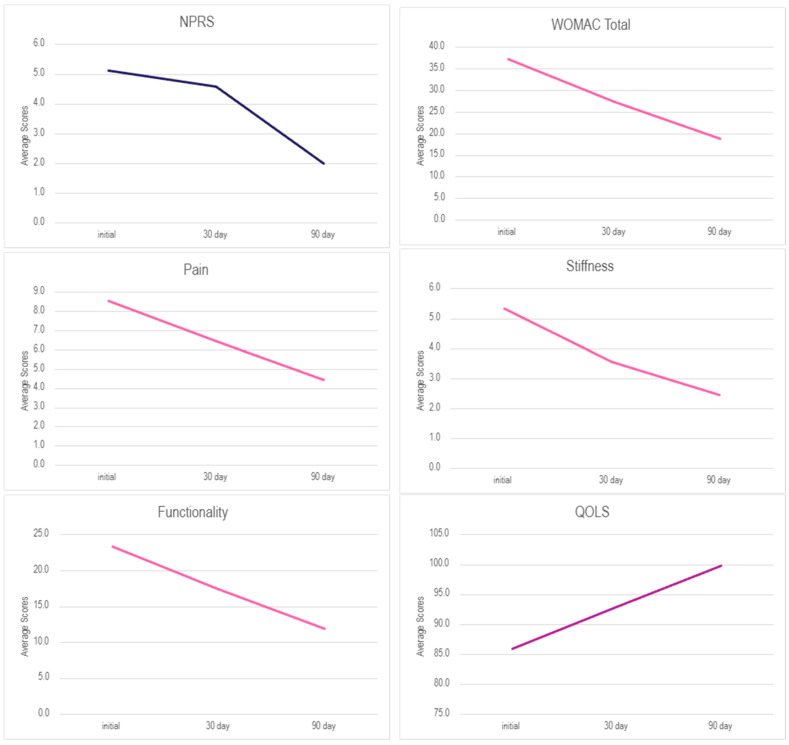
Average score over time for each scale.

When interpreting the results of the observational study, the decrease in WOMAC and NPRS scores equates to improvement, and the increase in QOLS scores equates to improvement. All nine patients received one application of Wharton’s jelly (WJ) and reported reductions in total WOMAC scores. Six out of seven patients reported a decrease in NPRS scores, and five out of eight patients reported higher QOLS scores. The percentage improvement for each scale in Table 2 equates to the percentage change from initial to final visits. The patients were further divided to identify if there was any correlation between male and female patients. Females had greater improvement in pain (NPRS) and quality of life (QOLS), while males had greater improvement in WOMAC scores (Table 3). Table 4 displays the average percentage of improvement (percentage of change in scores) between the two genders. Due to the skewness revealed in the descriptive statistics, nonparametric testing was utilized (Table 5). The Wilcoxon Signed-Rank test (WSRT) was conducted to determine any significant differences between initial and follow-up visits and confirmed the positive trend of improvements in scores (Table 6). A Bonferroni adjustment of the *p*-value was corrected to *p* < 0.0167 (adjusted α = 0.05/3). The test revealed a significant difference between initial and final scores for all WOMAC subscales and total (Table 6). The WOMAC total initial score (Md = 30, *n* = 9) and final score (Md = 11, *n* = 9), z = −2.668, *p* = 0.008, with a strong effect size, r = −0.63. The pain initial score (Md = 7, *n* = 9) and final score (Md = 2, *n* = 9), z = −2.68, *p* = 0.007, with a strong effect size, r = −0.63. The initial stiffness score (Md = 6, *n* = 9) and final score (Md = 2, *n* = 9), z = −2.53, *p* = 0.011, with a strong effect size, r = −0.60. Finally, the physical function initial score (Md = 19, *n* = 9) and final score (Md = 4, *n* = 9), z = −2.55, *p* = 0.011, with a strong effect size, r = −0.60. Figure 2 is the visual representation of the mean scores over time on each scale. A bootstrapped linear regression was conducted on demographic factors, including age, BMI, and gender, to predict total WOMAC improvement in the cohort. The results from BMI were excluded due to missing data in multiple data sets. Gender was not a significant predictor of total WOMAC improvement, as shown in Table 7. Age was determined to be a significant predictor of overall WOMAC improvement. Specifically, younger patients demonstrated greater improvement in WOMAC scores compared with older patients (*B* = −0.916, *p* = 0.028) (Table 8).

## 4. Discussion

The results reported in this study demonstrate the promising benefits of applying 150 mg WJ tissue allograft to supplement tissue defects associated with plantar fasciopathy. Individuals in the cohort reported positive outcomes on all three scales utilized, indicating progress in pain relief, decreased stiffness, and increased functionality (Figure 2, Table 2 and Table 3). Patients reported an average 3.1-point reduction in overall NPRS scores and an average 18.4-point reduction in total WOMAC scores from the initial application to the final follow-up date, which is a 60.98% and 49.55% improvement, respectively. An average 5.8-point improvement in the QOLS was also recorded between the initial visit and the 90-day follow-up, a 6.83% improvement. The results from the Wilcoxon Signed Rank Test (WSRT) revealed statistically significant improvements and strong effect sizes for the WOMAC total and subscales from initial to final visits, indicating meaningful clinical outcomes in patients undergoing WJ tissue allograft supplementation (Table 5 and Table 6). Table 3 and Table 4 displayed acute differences between male and female patients’ average scores across all six measures. Females had higher average NPRS and QOLS improvement, while males had higher average WOMAC improvement, and all patients reported a mean improvement in all scales. To check for statistical significance, a bootstrapped linear regression was performed and revealed no significant associations between total WOMAC improvement and gender. These findings suggest that incorporating sex-stratified analyses in larger patient pools can evaluate the importance, if any, of sex factors and provide valuable insight into personalized intervention protocols.

Bootstrapped linear regression was conducted on additional demographic factors such as age and BMI. The linear regression model reported statistically significant associations between age and overall total WOMAC improvement, demonstrating that younger patients experienced greater improvement in WOMAC outcomes compared with older patients. This finding aligns with previous studies suggesting that younger individuals may recover more effectively due to greater structural and biological strength than older patients [24,25,26]. While BMI associations were also calculated, no significance could be determined due to the missing BMI entries for three out of nine patients, limiting the sample size. Continued research on demographic factors is warranted to provide validation of these preliminary associations to support personalized management for patients with plantar fascia defects refractory to care. The results from this study provide a baseline of successful patient outcomes that should be confirmed by a prospective randomized controlled trial (RCT). A large-scale study could confirm the statistical significance observed and address some of the limitations presented by observational research. One limitation was the use of the WOMAC scale, which is traditionally utilized in hip and knee osteoarthritis, but is not routinely used in the assessment of PF. While the WOMAC is not validated for foot applications, the questions encompass functional issues that, when answered by the patient with the context of plantar fasciopathy, are still relevant. For example, pain when bearing weight, standing, walking, stair climbing, at night in bed, and putting on socks, along with stiffness throughout the day. Assessment tools frequently used in observing PF cases include the Visual Analog Scale (VAS) or NPRS for pain intensity, the Foot Function Index (FFI) for measuring pain, disability, and activity, and the American Orthopedic Foot and Ankle Society (AOFAS) score for examining the general functional status of the foot and ankle [27,28,29]. Potential sources of bias may be present, including the use of patient-reported outcome measures, which introduce minor variability in scores due to subjective interpretation. Pain subjectivity may be mitigated by the addition of before and after imaging to calculate improvement in tissue structure after WJ supplementation. Confounding variables, such as patient age and BMI, may influence outcomes and limit the generalizability of the findings to the broader population. Randomized controlled trials with larger patient pools that use a specific assessment scale would mitigate these limitations and strengthen the evidence for the efficacy of WJ in this specific application.

Favorable outcomes reported in this study align with the current literature on the application of WJ to other structural sites of defects. The structural profile of WJ is predominantly composed of collagen types I, III, and V, with additional beneficial compounds such as cytokines, growth factors, and hyaluronic acid, demonstrating functional compatibility in musculoskeletal tissue supplementation [22]. Tissue allografts used in this study have been imaged previously with scanning electron microscopy (SEM) to confirm the integrity of collagen structures present post-processing [30]. Cross-linking collagen fibers preserved through minimal manipulation practices make WJ tissue allografts clinically applicable to collagen-based structural tissues in the plantar fascia. The structural profile of plantar fascia contains two different kinds of tissues: a loose connective tissue sheath surrounding a tightly bundled collagen core [31]. The plantar fascia core predominantly contains type I cross-linking collagen fibers [31,32]. The collagenic ECM component in both WJ and plantar fascia functions to supply tensile strength and structural support in their corresponding anatomical regions. The similarities between the structure of the plantar fascia and WJ confirm the homologous nature of the tissues. Collagen breakdown of the plantar fascia, whether from excessive stress or natural causes, increases the disorganization of collagen fibers in the fascia, which decreases the strength and elasticity of the structural fibers [6]. The use of WJ tissue allografts in the plantar fascia directly replaces the damaged collagen matrix, mirroring existing scaffolding.

This observational research suggests that Wharton’s jelly is a promising alternative approach to current care methods. Standard-of-care procedures for plantar fasciopathy have been associated with various risks, complications, and inconsistent data results. PRP is one of the most popular alternative care protocols; however, the physicians involved in this study noticed inconsistent results using PRP on their other patients compared to the results reported by the WJ recipients. The varying outcomes associated with PRP could be attributed to dependence on the platelet counts and individual health conditions of the patients. A 2023 study by Rossi et al. reported significant differences in PRP therapy influenced by the patient’s platelet count [33]. Inconsistent protocols associated with PRP have contributed to the discrediting of many cases, mainly due to the neglect of evaluating patients’ platelet levels before therapy is administered [34]. WJ demonstrates effective and consistent results in this study, corroborating findings reported in other homologous use sites [23]. A study by Lai (2024) demonstrated that patients suffering from rotator cuff tears significantly improved in pain and range of motion with the application of WJ [23]. A 2025 study with before and after imaging highlights that the application of WJ may mitigate significant defects in the articular cartilage scaffold of the knee [35]. Future research will contribute to the growing pool of literature to strengthen support for the clinical potential of WJ tissue allografts in plantar fasciopathy.

## 5. Conclusions

All patients in this observational study received a single application of Wharton’s jelly (WJ) tissue allograft and experienced significant reductions in pain, stiffness, and physical function over a 90-day period. No adverse events were reported. The significant improvements observed in NPRS and WOMAC scales are encouraging; however, the small sample size, reliance on the WOMAC scale instead of a site-specific scale, and the absence of a control group prevent definitive conclusions from being established regarding the clinical outcomes of WJ tissue supplementation in plantar fascia degeneration. This pilot investigation offers preliminary results to support the feasibility and safety of WJ tissue allografts for patients with treatment-resistant plantar fasciopathy. Future studies are encouraged to confirm these exploratory findings and advocate for the inclusion of WJ in conservative care.

## Figures and Tables

**Table 1:** Patient age, Body Mass Index (BMI), and Gender.

Age Range		BMI Range	
20–29	0	Underweight (<18.5)	0
30–39	0	Healthy weight (18.5–24.9)	1
40–49	0	Overweight (25.0–29.9)	2
50–59	1	Obese (>30.0)	2
60–69	1	NA *	3
70–79	4	Mean BMI	30.9
80–89	2	**Gender**	
90–99	0	Male	5
Mean Age	73	Female	4

* NA (Not Applicable)—patients who were missing/did not report a BMI.

**Table 2:** Percentage of improvement for each scale from initial to follow-up visits.

Scale	Initial—Day 30	Initial—Day 90
NPRS	10.80%	60.98%
WOMAC Total	26.27%	49.55%
Pain	24.68%	48.05%
Stiffness	33.33%	54.17%
Functionality	25.24%	49.05%
QOLS	8.01%	6.83%

Key: NPRS: Numerical Pain Rating Scale, QOLS: Quality of life scale.

**Table 3:** Average scores and patient count for male (M) and female (F) patients across each scale.

Scale (Females)	*n*	Initial	*n*2	30 Day	*n*3	90 Day	Scale (Males)	*n*	Initial	*n*2	30 Day	*n*3	90 Day
NPRS	3	6.33	3	5	3	2.33	NPRS	5	4.4	4	4.25	5	1.8
WOMAC Total	4	48.25	4	36.25	4	26	WOMAC Total	5	28.4	5	20.4	5	13
Pain	4	11.5	4	9.75	4	6.75	Pain	5	6.2	5	3.8	5	2.6
Stiffness	4	6	4	3.75	4	3	Stiffness	5	4.8	5	3.4	5	2
Functionality	4	30.75	4	22.75	4	16.25	Functionality	5	17.4	5	13.2	5	8.4
QOLS	4	70.5	4	77.75	4	81.25	QOLS	5	98.4	5	105	4	102.5

Key: NPRS: Numerical Pain Rating Scale, QOLS: Quality of life scale.

**Table 4:** Percentage of improvement for male (M) and female (F) patients across each scale from initial to follow-up visits.

Scale (Females)	Initial—30 Day	Initial—90 Day	Scale (Males)	Initial—30 Day	Initial—90 Day
NPRS	21%	63%	NPRS	3%	59%
WOMAC Total	25%	46%	WOMAC Total	28%	54%
Pain	15%	41%	Pain	39%	58%
Stiffness	38%	50%	Stiffness	29%	58%
Functionality	26%	47%	Functionality	24%	52%
QOLS	10%	15%	QOLS	7%	4%

Key: NPRS: Numerical Pain Rating Scale, QOLS: Quality of life scale.

**Table 5:** Sample size and descriptive statistics for six scales at each interval.

		P2	S2	PF2	W2	Q2	FNPRS		FP
*n*	Valid	9	9	9	9	9	8		9
	Missing	0	0	0	0	0	1		0
Mean		6.4444	3.5556	17.4444	27.4444	92.8889	2.0000		4.4444
Median		4.0000	4.0000	12.0000	19.0000	95.0000	0.5000		2.0000
Std. Deviation		5.91843	2.96273	17.29242	24.92544	15.90161	2.87849		4.63980
Skewness		0.197	−0.220	0.702	0.480	0.086	1.533		0.534
Std. Error of Skewness		0.717	0.717	0.717	0.717	0.717	0.752		0.717
Kurtosis		−2.224	−1.786	−1.130	−1.548	−1.846	1.982		−1.469
Std. Error of Kurtosis		1.400	1.400	1.400	1.400	1.400	1.481		1.400
Minimum		0.00	0.00	1.00	1.00	74.00	0.00		0.00
Maximum		14.00	7.00	46.00	66.00	112.00	8.00		12.00
		**FS**		**FPF**		**FW**		**FQ**	
*n*	Valid	9		9		9		8	
	Missing	0		0		0		1	
Mean		2.4444		11.8889		18.7778		91.8750	
Median		2.0000		4.0000		11.0000		95.0000	
Std. Deviation		2.24227		15.14467		21.63202		18.23997	
Skewness		0.308		0.839		0.754		−0.672	
Std. Error of Skewness		0.717		0.717		0.717		0.752	
Kurtosis		−1.227		−1.472		−1.478		−1.064	
Std. Error of Kurtosis		1.400		1.400		1.400		1.481	
Minimum		0.00		0.00		0.00		65.00	
Maximum		6.00		34.00		51.00		112.00	

Key: NPRS: Numerical Pain Rating Scale, P: WOMAC—Pain, S: WOMAC—Stiffness, PF: WOMAC—Physical Function, W: WOMAC—Total, Q: Quality of life scale, 1: Initial Visit, 2: 30-Day Visit, 3: 90-Day Visit.

**Table 6:** Results from the Wilcoxon signed rank test; significant *p*-values are in bold.

Test Statistics ^a,d^						
	NPRS2—NPRS1	P2—P1	S2—S1	PF2—PF1	W2—W1	Q2—Q1
Z	−0.736 ^b^	−1.442 ^b^	−2.032 ^b^	−1.014 ^b^	−1.120 ^b^	−1.521 ^c^
Asymp. Sig. (2-tailed)	0.461	0.149	0.042	0.310	0.263	0.128
r						
	**FNPRS—NPRS2**	**FP—P2**	**FS—S2**	**FPF—PF2**	**FW—W2**	**FQ—Q2**
Z	−1.761 ^b^	−2.154 ^b^	−1.200 ^b^	−2.524 ^b^	−2.492 ^b^	−0.140 ^c^
Asymp. Sig. (2-tailed)	0.078	0.031	0.230	**0.012**	**0.013**	0.889
r				**0.42**	**0.42**	
	**FNPRS—NPRS1**	**FP—P1**	**FS—S1**	**FPF—PF1**	**FW—W1**	**FQ—Q1**
Z	−2.124 ^b^	−2.680 ^b^	−2.536 ^b^	−2.547 ^b^	−2.668 ^b^	−1.016 ^c^
Asymp. Sig. (2-tailed)	0.034	**0.007**	**0.011**	**0.011**	**0.008**	0.310
r		**0.60**	**0.63**	**0.63**	**0.60**	

Key: NPRS: Numerical Pain Rating Scale, P: WOMAC—Pain, S: WOMAC—Stiffness, PF: WOMAC—Physical Function, W: WOMAC—Total, Q: Quality of life scale, 1: Initial Visit, 2: 30-Day Visit, 3: 90-Day Visit, ^a^ Wilcoxon Signed Ranks Test, ^b^ Based on positive ranks, ^c^ Based on negative ranks, ^d^ Adjusted for multiple comparisons: Bonferroni.

**Table 7:** Bootstrapped linear regression analysis of gender for WOMAC improvement.

Bootstrap for Coefficients							
Model		B	Bootstrap ^a^				
			Bias	Std. Error	Sig. (2-Tailed)	BCa 95% Confidence Interval	
						Lower	Upper
1	(Constant)	−29.100	0.085 ^b^	23.433 ^b^	0.389 ^b^	−99.375 ^b^	5.550 ^b^
	Gender	6.850	0.000 ^b^	12.456 ^b^	0.624 ^b^	−11.333 ^b^	30.333 ^b^

^a^ Unless otherwise noted, bootstrap results are based on 2000 bootstrap samples or ^b^ based on 1994 samples.

**Table 8:** Bootstrapped linear regression analysis of age for WOMAC improvement.

Bootstrap for Coefficients							
Model		B	Bootstrap ^a^				
			Bias	Std. Error	Sig. (2-Tailed)	BCa 95% Confidence Interval	
						Lower	Upper
1	(Constant)	53.234	1.033	18.897	0.039	−2.540	120.909
	Age	−0.916	−0.010	0.259	**0.028**	−1.280	−0.601

^a^ Unless otherwise noted, bootstrap results are based on 2000 bootstrap samples.

## Data Availability

Data are available upon request.
